# Association of grand multiparity with adverse birth outcomes and sociodemographic characteristics: an analysis of nationwide birth data in Japan

**DOI:** 10.1186/s12978-025-02246-0

**Published:** 2025-12-27

**Authors:** Tasuku Okui, Naoki Nakashima

**Affiliations:** 1 Medical Information Center, Kyushu University Hospital, 3-1-1 Maidashi, Higashi-ku, Fukuoka, 812-8582 Japan; 2 Department of Medical Informatics, Faculty of Medical Sciences, Kyushu University, Fukuoka, Japan

**Keywords:** Japan, Parity, Grand multiparity, Socioeconomic status, Vital statistics

## Abstract

**Background:**

In Japan, grand multiparity’s associations with representative adverse birth outcomes such as preterm birth and low birth weight and the sociodemographic features of grand multiparity have not been investigated yet. We investigated these using nationwide live birth data.

**Methods:**

Live birth data of the Vital Statistics: Occupational and Industrial Aspects in Japan in 2010, 2015, and 2020 were used. Parity was categorized into primiparous (1 delivery), non-grand multiparous (2–4 deliveries), and grand multiparous (≥ 5 deliveries). Preterm birth (< 37 gestational weeks), low birth weight (< 2,500 g), macrosomia (≥ 4,000 g), small-for-gestational-age infant (weighing less than the 10th percentile for gestational age), and large-for-gestational-age infant (weighing more than the 90th percentile for gestational age) were considered adverse birth outcomes, while the parental age groups, parental nationalities, parental occupations, and household occupation were considered sociodemographic characteristics. A log-binomial regression model was used to investigate the association between grand multiparity and the chosen adverse birth outcomes, as well as its sociodemographic features.

**Results:**

In total, 2,623,696 singleton live births within marriage were used for the analysis. The proportions of preterm birth, low birth weight, macrosomia, and large-for-gestational-age infant for grand multiparous women were higher than those of primiparous and non-grand multiparous women, and grand multiparity was significantly associated with a higher risk of those adverse birth outcomes. In addition, a non-Japanese mother was significantly associated with a higher prevalence of grand multiparity, while households with a full-time worker at a larger company and upper non-manual workers had a significantly lower prevalence of grand multiparity than the other household occupations and parental occupations, respectively.

**Conclusions:**

Grand multiparity was significantly associated with a higher risk of adverse birth outcomes. In addition, sociodemographic characteristics such as maternal nationality and parental occupations were shown to be associated with grand multiparity.

**Supplementary Information:**

The online version contains supplementary material available at 10.1186/s12978-025-02246-0.

## Background

High parity is known to be associated with various adverse health outcomes worldwide, and it was shown to be associated with higher all-cause mortality risk in a meta-analysis [1]. In Japan, associations between high parity and cardiovascular disease mortality and tooth loss have been demonstrated [2, 3]. Having at least five deliveries is often considered high parity in perinatal epidemiology, and it is called grand multiparity.

In Japan, the number of live births from women who were not grand multiparous has been decreasing over the years as a result of the declining birth rate, while the number of births from grand multiparous women has relatively stable in recent decades. As a result, the prevalence of the first-born infants decreased from 48.1% in 2005 to 46.1% in 2020, and that of the second-born infants decreased 37.5% in 2005 to 36.1% in 2020 [4]. In contrast, the prevalence of grand mutiparity among live births increased from 0.6% in 2005 to 1.1% in 2020 in Japan [4]. The prevalence of grand multiparity differed depending on countries, and the prevalence exceeded 10% in some studies conducted in developing countries [5–7]. In addition, the prevalence of grand multiparity was more than 7% in 1989–2000 in the Unites States [8], and the prevalence among multiparous women was 13.9% in Israel [9].

Grand multiparity was shown to be associated with a higher risk of adverse birth outcomes, such as preterm birth, low birth weight, macrosomia, small-for-gestational-age (SGA) infant, and large-for-gestational-age (LGA) infant worldwide [10–14]. In contrast, the association between grand multiparity and adverse birth outcomes varies among studies [15], and a meta-analysis demonstrated that grand multiparity was not associated with preterm birth or low birth weight [16]. In Japan, one study demonstrated that a previous experience of at least three live births was significantly associated with infant mortality compared with nulliparity [17]; however, associations between grand multiparity and representative adverse birth outcomes such as preterm birth and low birth weight have been investigated using nationwide data in Japan.

Some previous studies have investigated the characteristics of grand multiparous women in other countries. Sociodemographic profiles of grand multiparous women were investigated in the United Arab Emirates [18], and factors such as higher maternal age and pre-pregnancy overweight were shown to be associated with it. In addition, a study conducted in Nigeria demonstrated that higher levels of education and higher household wealth reduced the likelihood of grand multiparity among women [19]. No study had investigated the sociodemographic characteristics of parents with grand multiparity in Japan, and it is important to investigate the characteristics associated with grand multiparity if it is associated with adverse birth outcomes.

In this study, we aimed to investigate the associations of grand multiparity with adverse birth outcomes and sociodemographic characteristics using nationwide live birth data in Japan.

## Methods

### Data and data processing

We used live birth data in the Vital Statistics: Occupational and Industrial Aspects in Japan in 2010, 2015, and 2020. The Vital Statistics Survey investigating the occupations of individuals is conducted every 5 fiscal years. The data were provided by the Ministry of Health, Labour and Welfare and the National Statistics Center in Japan on the basis of Article 33 of the Statistics Act, and we used an on-site facility for the official statistics data to analyze the data. The data cover all live births that were notified to local governments in Japan. We used information on birthweight, gestational age, number of fetuses, and sex of infants, along with marital status, occupation, parity, age, and nationality of their mothers. In addition, information on paternal nationality, paternal age, paternal occupation, and household occupation was also used. Household occupation indicates the type of occupation or employment status of the top earner of the household, and it consists of farmer, self-employed, full-time worker at a smaller company (full-time worker at a company with < 100 employees), full-time worker at a larger company (public servants, board member of a company, and full-time workers at a company with ≥ 100 employees), other workers, and unemployed. Parity in the data indicates the sum of the number of fetal deaths that occurred at a gestational age of ≥ 22 completed weeks and the number of live births.

We used preterm birth, low birth weight, macrosomia, SGA infant, and LGA infant as the adverse birth outcomes. Preterm birth was defined as infants born before 37 completed weeks of gestation. Low birth weight was defined as infants weighing < 2,500 g, and macrosomia was defined as a birth weight of ≥ 4,000 g. SGA infant and LGA infant were defined based on the anthropometric chart for neonates in Japan [20]. Specifically, SGA infants and LGA infants were defined as infants whose birthweights were less than the 10th percentile and greater than the 90th percentile for each combination of gestational age, sex, and parity (primiparity and multiparity), respectively, and infants whose birthweights were within the 10th − 90th percentile were defined as appropriate-for-gestational-age infants. The definition of grand multiparity slightly varied from one study to the next. In this study, grand multiparity was defined as five or more deliveries [16, 21–23], which include the delivery of this time, and non-grand multiparous women were defined as those with 2–4 deliveries.

There were 13 different parental occupations in the data, and those were categorized into occupational classes for ease of interpretation. Specifically, those were classified into upper non-manual workers (administrative and managerial workers; professional and engineering workers), lower non-manual workers (clerical workers; sales workers; and service workers), manual workers (manufacturing process workers; transport and machine operating workers; construction and mining workers; and carrying, cleaning, packaging, and related workers), other workers (security workers; agriculture, forestry, and fishery workers; and workers in unclassifiable occupations), and unemployed persons. The other workers and unemployed households in the household occupation were categorized as others in order to avoid multicollinearity in the analysis. Maternal and paternal age groups were categorized into the following age groups: less than 25 years, 25–29 years, 30–34 years, 35–39 years, and ≥ 40 years. Parental nationalities were categorized into Japanese and non-Japanese.

Data on parental ages, nationalities, occupations, and household occupation were used as sociodemographic characteristics. The above-mentioned parental characteristics were employed because they are related to both adverse birth outcomes and parity. In addition, those were the parental characteristics that were available in the data. Specifically, maternal and paternal ages were used because they are risk factors of adverse birth outcomes and are associated with the prevalence of grand multiparity [10, 24, 25]. The household occupation and parental occupations were used as socioeconomic characteristics of parents, and socioeconomic status is known to affect both adverse birth outcomes and grand multiparity [26–28]. The household occupation was used in addition to maternal and paternal occupations because the household occupation consists of categories which are relevant to employment status. Therefore, the household occupation reflects different aspects of socioeconomic status from parental occupations. Maternal and paternal nationalities were also used because those were shown to be associated with adverse birth outcomes in Japan [29, 30], and it is also possible that the prevalence of grand multiparity differs depending on parental nationalities.

We used singleton live births in the analysis, and live births within marriage were used in the analysis because we wanted to use paternal characteristics in the analysis. Moreover, infants born within 22–41 weeks of gestation were used in the analysis for SGA infant and LGA infant because the anthropometric chart in Japan covers only those gestational weeks.

### Statistical analysis

We counted the number of births by parity and birth characteristics, and the prevalence of grand multiparity was calculated by birth characteristics. In addition, a log-binomial regression model was used to investigate the association between grand multiparity and adverse birth outcomes, and crude and adjusted analyses were conducted. Each of the adverse birth outcomes was used as an outcome variable. Only parity was used in the crude analysis, while parity, parental age groups, parental nationalities, parental occupations, household occupation, and birth year were included as explanatory variables in the adjusted analysis. A common identification number does not exist for the same parents across different time points in the data, and sandwich variance was used in the regression analyses in order to deal with potential overlaps of parents in the birth data across different years. The measure of association used in this study was the risk ratio (RR) with its 95% confidence interval (CI), and the p-value was calculated for every association. In addition, from the prevalence of each adverse birth outcome among non-grand multiparous women and the adjusted RR, the absolute risk difference between grand multiparous women and non-grand multiparous women was calculated for each adverse birth outcome. Moreover, a log-binomial regression model was also used to investigate the association between sociodemographic characteristics and grand multiparity. Grand multiparity, which was binary variable in this analysis, was used as the outcome variable, and parental age groups, parental nationalities, parental occupations, household occupation, and birth year were included as explanatory variables. In addition to crude analyses using each of the explanatory variables separately, an adjusted analysis using all the explanatory variables was conducted. The prevalence ratio (PR), 95% CI, and p-value were calculated for each of the explanatory variables. A p-value of less than 0.05 was considered statistically significant. In the adjusted regression analyses, multicollinearity was assessed by generalized variance inflation factor (GVIF) [31], and the adjusted GVIF, that is, GVIFˆ(1/(2×Degrees of freedom)) was used as the criterion. The adjusted GVIF larger than 2 was often used as the criterion of multicollinearity [32–34] and was also used as the criterion in this study.

Births with missing entries for the variables were not used, and a complete-case analysis was conducted. Supplementary Table 1 shows the proportion of missing data for each variable, and the proportion of missing data in total was 6.3%. In contrast, a multiple imputation analysis using multivariate imputation by chained equations was also conducted as a sensitivity analysis for the adjusted regression analyses [35]. The number of imputations was 10, and predictive mean matching was used as the imputation method. All the statistical analyses were performed using R4.4 [36], with car, lmtest, mice, and sandwich used as packages. The statistics shown in this study were created by the authors using data that were provided by the Ministry of Health, Labour and Welfare and the National Statistics Center, and they are not statistics published by the Ministry.

## Results

**Fig. 1:**
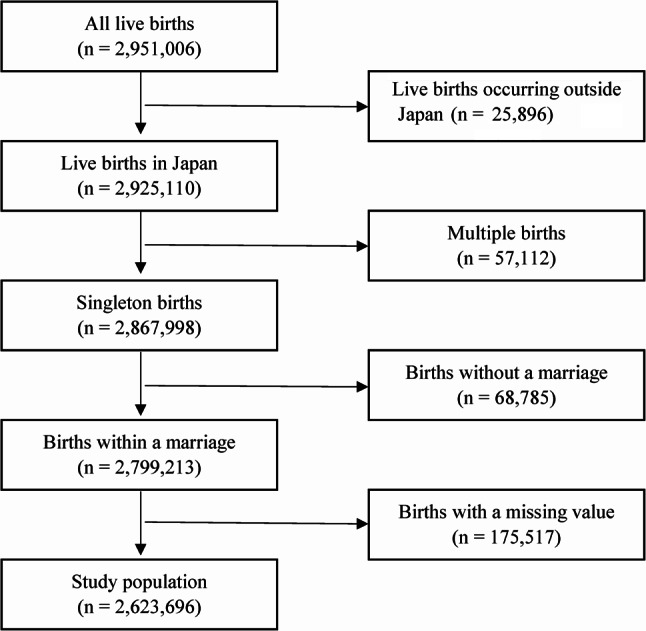
Flowchart of the selection process of the study population

Figure 1 shows the flowchart of the population selection process. After excluding 25,896 births occurring outside Japan, 57,112 multiple births, 68,785 births without a marriage, and 175,517 births with a missing value, a total of 2,623,696 live births were used for the analysis.

Table 1 shows the number (%) of births for each parity and the prevalence of grand multiparity by birth characteristics. The overall prevalence of grand multiparity was 0.84%. The prevalence of grand multiparity increased with parental ages, and this prevalence was higher among non-Japanese parents than among Japanese parents. Among the household occupations, the prevalence of grand multiparity for households with at least one full-time worker at a larger company was the lowest, and that for self-employed households was the highest. Moreover, the prevalence of grand multiparity increased progressively from upper non-manual workers to lower non-manual workers and then to manual workers according to parental occupations, and the prevalence for unemployed persons was the highest among paternal occupations. Furthermore, the prevalence of grand multiparity increased from 2010 to 2020.

**Table 1:** Births (%) for each parity and the prevalence of grand multiparity by birth characteristics

Characteristics	Primiparous^1^	Non-grand multiparous^1^	Grand multiparous^1^	Prevalence of grand multiparity
	Number (%)	Number (%)	Number (%)	%
Total	1,237,001 (100.0)	1,364,777 (100.0)	21,918 (100.0)	0.84
Maternal age group				
24 years or less	169,703 (13.7)	76,054 (5.6)	160 (0.7)	0.07
25–29 years	419,804 (33.9)	292,592 (21.4)	2,197 (10.0)	0.31
30–34 years	404,951 (32.7)	546,525 (40.0)	6,991 (31.9)	0.73
35–39 years	197,105 (15.9)	374,762 (27.5)	8,963 (40.9)	1.54
40 years or more	45,438 (3.7)	74,844 (5.5)	3,607 (16.5)	2.91
Paternal age group				
24 years or less	112,217 (9.1)	48,481 (3.6)	268 (1.2)	0.17
25–29 years	341,733 (27.6)	219,058 (16.1)	1,869 (8.5)	0.33
30–34 years	403,148 (32.6)	474,547 (34.8)	5,327 (24.3)	0.60
35–39 years	241,961 (19.6)	407,677 (29.9)	7,319 (33.4)	1.11
40 years or more	137,942 (11.2)	215,014 (15.8)	7,135 (32.6)	1.98
Maternal nationality				
Japanese	1,212,142 (98.0)	1,339,104 (98.1)	21,175 (96.6)	0.82
Non-Japanese	24,859 (2.0)	25,673 (1.9)	743 (3.4)	1.45
Paternal nationality				
Japanese	1,211,255 (97.9)	1,340,202 (98.2)	21,322 (97.3)	0.83
Non-Japanese	25,746 (2.1)	24,575 (1.8)	596 (2.7)	1.17
Household occupation^2^				
Farmer	14,746 (1.2)	22,017 (1.6)	510 (2.3)	1.37
Self-employed	78,889 (6.4)	110,244 (8.1)	3,517 (16.0)	1.83
Full-time worker at a smaller company	412,496 (33.3)	477,707 (35.0)	8,728 (39.8)	0.97
Full-time worker at a larger company	602,096 (48.7)	619,105 (45.4)	5,816 (26.5)	0.47
Others	128,774 (10.4)	135,704 (9.9)	3,347 (15.3)	1.25
Maternal occupation^3^				
Upper non-manual workers	224,799 (18.2)	209,896 (15.4)	1,920 (8.8)	0.44
Lower non-manual workers	326,169 (26.4)	299,354 (21.9)	4,864 (22.2)	0.77
Manual workers	27,789 (2.2)	33,668 (2.5)	841 (3.8)	1.35
Other workers	25,964 (2.1)	28,400 (2.1)	541 (2.5)	0.99
Unemployed persons	632,280 (51.1)	793,459 (58.1)	13,752 (62.7)	0.96
Paternal occupation^3^				
Upper non-manual workers	383,803 (31.0)	396,795 (29.1)	4,474 (20.4)	0.57
Lower non-manual workers	448,460 (36.3)	459,459 (33.7)	5,377 (24.5)	0.59
Manual workers	292,142 (23.6)	384,219 (28.2)	9,470 (43.2)	1.38
Other workers	92,121 (7.4)	108,461 (7.9)	1,718 (7.8)	0.85
Unemployed persons	20,475 (1.7)	15,843 (1.2)	879 (4.0)	2.36
Infant sex				
Female	602,245 (48.7)	665,291 (48.7)	10,641 (48.5)	0.83
Male	634,756 (51.3)	699,486 (51.3)	11,277 (51.5)	0.84
Birth year (fiscal year)				
2010	451,509 (36.5)	494,146 (36.2)	7,029 (32.1)	0.74
2015	430,036 (34.8)	470,689 (34.5)	7,265 (33.1)	0.80
2020	355,456 (28.7)	399,942 (29.3)	7,624 (34.8)	1.00

^1^Parity was categorized into primiparous (1 delivery), non-grand multiparous (2–4 deliveries), and grand multiparous (≥ 5 deliveries)

^2^Household occupation indicates the type of occupation or employment status of the top earner of the household. The full-time worker at a smaller company indicates a full-time worker at a company with < 100 employees, and the full-time worker at a larger company indicates public servants, board member of a company, and full-time workers at a company with ≥ 100 employees

^3^Parental occupations were classified into upper non-manual workers (administrative and managerial workers; professional and engineering workers), lower non-manual workers (clerical workers; sales workers; and service workers), manual workers (manufacturing process workers; transport and machine operating workers; construction and mining workers; and carrying, cleaning, packaging, and related workers), other workers (security workers; agriculture, forestry, and fishery workers; and workers in unclassifiable occupations), and unemployed persons in the analysis

Table 2 shows the number and prevalence of each adverse birth outcome by parity. The proportions of preterm birth, low birth weight, macrosomia, and LGA infant in grand multiparous women were higher than those in primiparous and non-grand multiparous women, while the proportion of SGA infant was comparable among the three types of parity.

**Table 2:** Number and prevalence of each adverse birth outcome by parity

**Parity** ^1^	Preterm birth^2^	Low birth weight^2^	Macrosomia^2^	SGA^2^	LGA^2^
	**Number (%)**	**Number (%)**	**Number (%)**	**Number (%)**	**Number (%)**
Primiparous	55,232 (4.5)	109,889 (8.9)	8,244 (0.7)	91,711 (7.4)	140,695 (11.4)
Non-grand multiparous	64,221 (4.7)	99,628 (7.3)	13,450 (1.0)	97,173 (7.1)	130,987 (9.6)
Grand multiparous	1,704 (7.8)	2,176 (9.9)	431 (2.0)	1,599 (7.3)	3,005 (13.7)

*SGA* small-for-gestational-age, *LGA* large-for-gestational-age

^1^Parity was categorized into primiparous (1 delivery), non-grand multiparous (2–4 deliveries), and grand multiparous (≥ 5 deliveries). ^2^Preterm birth was defined as infants born before 37 completed weeks of gestation. Low birth weight was defined as infants weighing < 2,500 g, and macrosomia was defined as a birth weight of ≥ 4,000 g. SGA infants and LGA infants were defined as infants whose birthweights were less than the 10th percentile and greater than the 90th percentile for each combination of gestational age, sex, and parity (primiparity and multiparity), respectively

Table 3 shows the results of the regression analyses investigating the associations between parity and adverse birth outcomes. Similar results were obtained for crude and adjusted analysis. The adjusted RRs of grand multiparous women to non-grand multiparous women for preterm birth, low birth weight, macrosomia, and LGA infant were 1.45 (95% CI: 1.39 1.52), 1.24 (95% CI: 1.19, 1.29), 1.77 (95% CI: 1.61, 1.95), and 1.33 (95% CI: 1.28, 1.37), respectively, and all these associations were statistically significant. In addition, the maximum value of the adjusted GVIF among explanatory variables was 1.32 in the analyses. Moreover, the absolute risk differences between grand multiparous women and non-grand multiparous women were derived from the prevalence of non-grand multiparous women and the point estimates of the adjusted RRs, and those for preterm birth, low birth weight, macrosomia, and LGA infant were 2.1%, 1.8%, 0.8%, and 3.2%, respectively. Supplementary Table 2 shows the result of the adjusted regression analysis investigating the associations between parity and adverse birth outcomes using multiple imputation, and it was confirmed that the result was similar to that of the complete-case analysis.

**Table 3:** The result of regression analysis investigating an association between parity and adverse birth outcomes

	Crude analysis			Adjusted analysis	
Parity and adverse birth outcomes^1,2^	Crude RR (95% CI)	*p*-value		Adjusted RR (95% CI)^3^	*p*-value
Preterm birth					
Primiparous	0.95 (0.94, 0.96)	< 0.001		1.01 (1.00, 1.02)	0.120
Non-grand multiparous	1.00 (Reference)			1.00 (Reference)	
Grand multiparous	1.65 (1.58, 1.73)	< 0.001		1.45 (1.39, 1.52)	< 0.001
Low birth weight					
Primiparous	1.22 (1.21, 1.23)	< 0.001		1.28 (1.26, 1.29)	< 0.001
Non-grand multiparous	1.00 (Reference)			1.00 (Reference)	
Grand multiparous	1.36 (1.31, 1.42)	< 0.001		1.24 (1.19, 1.29)	< 0.001
Macrosomia					
Primiparous	0.68 (0.66, 0.69)	< 0.001		0.71 (0.69, 0.73)	< 0.001
Non-grand multiparous	1.00 (Reference)			1.00 (Reference)	
Grand multiparous	2.00 (1.81, 2.19)	< 0.001		1.77 (1.61, 1.95)	< 0.001
SGA					
Primiparous	1.04 (1.04, 1.05)	< 0.001		1.06 (1.05, 1.07)	< 0.001
Non-grand multiparous	1.00 (Reference)			1.00 (Reference)	
Grand multiparous	1.03 (0.98, 1.08)	0.302		0.99 (0.95, 1.04)	0.768
LGA					
Primiparous	1.19 (1.18, 1.20)	< 0.001		1.22 (1.21, 1.23)	< 0.001
Non-grand multiparous	1.00 (Reference)			1.00 (Reference)	
Grand multiparous	1.43 (1.38, 1.48)	< 0.001		1.33 (1.28, 1.37)	< 0.001

*RR* risk ratio, *CI* confidence interval, *SGA* small-for-gestational-age, *LGA* large-for-gestational-age

^1^Parity was categorized into primiparous (1 delivery), non-grand multiparous (2–4 deliveries), and grand multiparous (≥ 5 deliveries)

^2^Preterm birth was defined as infants born before 37 completed weeks of gestation. Low birth weight was defined as infants weighing < 2,500 g, and macrosomia was defined as a birth weight of ≥ 4,000 g. SGA infants and LGA infants were defined as infants whose birthweights were less than the 10th percentile and greater than the 90th percentile for each combination of gestational age, sex, and parity (primiparity and multiparity), respectively

^3^Parental age groups, parental nationalities, household occupation, parental occupations, and birth year were adjusted

Table 4 shows the results of the regression analysis investigating the sociodemographic characteristics of grand multiparity. There was a statistically significant association between parental ages and grand multiparity. Specifically, adjusted PRs for 35–39 years and ≥ 40 years among mothers were 1.82 (95% CI: 1.75, 1.88) and 2.89 (95% CI: 2.76, 3.03), respectively, and those among fathers were 1.23 (95% CI: 1.18, 1.27) and 1.62 (95% CI: 1.55, 1.69), respectively. In addition, non-Japanese maternal status was significantly positively associated with grand multiparity, with an adjusted PR of 1.27 (95%CI: 1.17, 1.38). Regarding the household occupation, households with a farmer, a self-employed person, a full-time worker at a smaller company, and other occupations had a significantly higher prevalence of grand multiparity than those with a full-time worker at a larger company, and the adjusted PRs were 2.69 (95% CI: 2.46, 2.95), 3.11 (95% CI: 2.98, 3.24), 1.92 (95% CI: 1.86, 1.99), and 2.47 (95% CI: 2.36, 2.58), respectively. Regarding parental occupations, lower non-manual workers, manual workers, other workers, and unemployed persons had a significantly higher prevalence of grand multiparity than upper non-manual workers. Specifically, the adjusted PRs of those maternal occupations were 1.58 (95% CI: 1.50, 1.67), 2.02 (95% CI: 1.86, 2.19), 1.80 (95% CI: 1.62, 1.99), and 2.20 (95% CI: 2.09, 2.31), respectively, and those of the paternal occupations were 1.07 (95% CI: 1.03, 1.12), 2.53 (95% CI: 2.44, 2.62), 1.45 (95% CI: 1.37, 1.55), and 3.49 (95% CI: 3.24, 3.76), respectively. In addition, the birth year 2020 was significantly associated with a higher prevalence of grand multiparity, with the adjusted PR of 1.35 (95% CI: 1.31, 1.40). There was a statistically significant association between non-Japanese father and grand multiparity in the crude analysis, while it was not observed in the adjusted analysis. In addition, the maximum value of the adjusted GVIF among explanatory variables was 1.15. Supplementary Table 3 shows the result of the adjusted regression analysis investigating the sociodemographic characteristics of grand multiparity using multiple imputation, and it was confirmed that the result was similar to that of the complete-case analysis.

**Table 4:** The result of regression analysis investigating sociodemographic characteristics of grand multiparity

	Crude analysis		Adjusted analysis	
Characteristics	PR (95% CI)	*p*-value	Adjusted PR (95% CI)	*p*-value
Maternal age group				
24 years or less	0.09 (0.08, 0.10)	< 0.001	0.06 (0.05, 0.07)	< 0.001
25–29 years	0.42 (0.40, 0.44)	< 0.001	0.41 (0.39, 0.44)	< 0.001
30–34 years	1.00 (Reference)		1.00 (Reference)	
35–39 years	2.12 (2.05, 2.18)	< 0.001	1.82 (1.75, 1.88)	< 0.001
40 years or more	3.99 (3.84, 4.15)	< 0.001	2.89 (2.76, 3.03)	< 0.001
Paternal age group				
24 years or less	0.28 (0.24, 0.31)	< 0.001	1.08 (0.92, 1.25)	0.289
25–29 years	0.55 (0.52, 0.58)	< 0.001	0.94 (0.88, 0.99)	0.027
30–34 years	1.00 (Reference)		1.00 (Reference)	
35–39 years	1.85 (1.78, 1.91)	< 0.001	1.23 (1.18, 1.27)	< 0.001
40 years or more	3.28 (3.17, 3.40)	< 0.001	1.62 (1.55, 1.69)	< 0.001
Maternal nationality				
Japanese	1.00 (Reference)		1.00 (Reference)	
Non-Japanese	1.76 (1.64, 1.89)	< 0.001	1.27 (1.17, 1.38)	< 0.001
Paternal nationality				
Japanese	1.00 (Reference)		1.00 (Reference)	
Non-Japanese	1.41 (1.30, 1.53)	< 0.001	0.95 (0.87, 1.04)	0.282
Household occupation^1^				
Farmer	2.89 (2.64, 3.16)	< 0.001	2.69 (2.46, 2.95)	< 0.001
Self-employed	3.85 (3.69, 4.02)	< 0.001	3.11 (2.98, 3.24)	< 0.001
Full-time worker at a smaller company	2.05 (1.98, 2.12)	< 0.001	1.92 (1.86, 1.99)	< 0.001
Full-time worker at a larger company	1.00 (Reference)		1.00 (Reference)	
Others	2.64 (2.53, 2.75)	< 0.001	2.47 (2.36, 2.58)	< 0.001
Maternal occupation^2^				
Upper non-manual workers	1.00 (Reference)		1.00 (Reference)	
Lower non-manual workers	1.75 (1.66, 1.85)	< 0.001	1.58 (1.50, 1.67)	< 0.001
Manual workers	3.07 (2.83, 3.33)	< 0.001	2.02 (1.86, 2.19)	< 0.001
Other workers	2.24 (2.04, 2.46)	< 0.001	1.80 (1.62, 1.99)	< 0.001
Unemployed persons	2.17 (2.07, 2.28)	< 0.001	2.20 (2.09, 2.31)	< 0.001
Paternal occupation^2^				
Upper non-manual workers	1.00 (Reference)		1.00 (Reference)	
Lower non-manual workers	1.03 (0.99, 1.07)	0.107	1.07 (1.03, 1.12)	< 0.001
Manual workers	2.42 (2.34, 2.51)	< 0.001	2.53 (2.44, 2.62)	< 0.001
Other workers	1.49 (1.41, 1.58)	< 0.001	1.45 (1.37, 1.55)	< 0.001
Unemployed persons	4.15 (3.86, 4.45)	< 0.001	3.49 (3.24, 3.76)	< 0.001
Birth year (fiscal year)				
2010	1.00 (Reference)		1.00 (Reference)	
2015	1.08 (1.05, 1.12)	< 0.001	1.03 (1.00, 1.06)	0.084
2020	1.35 (1.31, 1.40)	< 0.001	1.35 (1.31, 1.40)	< 0.001

*PR* prevalence ratio, *CI* confidence interval

^1^Household occupation indicates the type of occupation or employment status of the top earner of the household. The full-time worker at a smaller company indicates a full-time worker at a company with < 100 employees, and the full-time worker at a larger company indicates public servants, board member of a company, and full-time workers at a company with ≥ 100 employees

^2^Parental occupations were classified into upper non-manual workers (administrative and managerial workers; professional and engineering workers), lower non-manual workers (clerical workers; sales workers; and service workers), manual workers (manufacturing process workers; transport and machine operating workers; construction and mining workers; and carrying, cleaning, packaging, and related workers), other workers (security workers; agriculture, forestry, and fishery workers; and workers in unclassifiable occupations), and unemployed persons in the analysis

## Discussion

This study demonstrated that grand multiparity was significantly associated with preterm birth, low birth weight, macrosomia, and LGA infant, and sociodemographic factors of parents such as parental occupations were shown to be associated with grand multiparity.

In previous studies, the results of investigating the associations between grand multiparity and adverse birth outcomes varied depending on studies [15, 16]. Similar to our study, some previous studies in other countries have demonstrated significant associations between grand multiparity and preterm birth, low birth weight, LGA infant, and macrosomia [10, 12–14, 37]. An advanced maternal age is a possible reason for the high prevalence of adverse birth outcomes among grand multiparous women. However, we adjusted maternal age groups in the adjusted regression analysis, and grand multiparity was still significantly associated with adverse birth outcomes. Although the reasons for the associations were not properly elucidated, the lower socioeconomic status of grand multiparous women was pointed out as a reason in previous study [38]. It was demonstrated in this study that households with at least one full-time worker at a larger company had a significantly lower prevalence of grand multiparity compared with the other households. In addition, upper non-manual workers had a lower prevalence of grand multiparity than the other types of occupations, and the PR was particularly high in unemployed fathers. These results suggested that grand multiparity was an indicator of lower socioeconomic status in Japan. Lower socioeconomic status was positively associated with adverse birth outcomes such as preterm birth and low birth weight in Japan [26, 39], and it is considered to be a reason for the association between grand multiparity and adverse birth outcomes.

In addition, grand multiparity was shown to be associated with some pregnancy complications, such as gestational diabetes, gestational hypertension, and placenta praevia [14, 40]. These factors can also explain the associations between grand multiparity and adverse birth outcomes. It is hypothesized that pregnancy induces inflammation, insulin resistance, and oxidative stress, all of which can affect endothelial function in blood vessels and predispose the patient to cardiovascular diseases—an effect that may increase with the number of pregnancies [41]. In addition, it has been demonstrated that repeated pregnancies or multiparity alters cardiac structures in women, and changes in left ventricular end-diastolic volume and left ventricular ejection fraction have been shown [42]. These adverse effects caused by repeated pregnancy might be a possible cause of the association between grand multiparity and pregnancy complications. In contrast, no significant association between SGA infant and grand multiparity was observed in this study, whereas a significant association between LGA infant and grand multiparity was demonstrated. In addition, the RR of grand multiparity for macrosomia was much larger than that for low birth weight. These results indicated that grand multiparity was particularly associated with an increase in birthweight rather than a decrease in birthweight in Japan. A high body mass index was shown to be a risk factor for LGA infant and macrosomia [43], and there are some studies that showed an association between grand multiparity and a high body mass index among women [44–46]. In addition, gestational diabetes, whose prevalence was shown to be high among grand multiparous women, was shown to be a major risk factor for macrosomia and LGA infant [47]. Therefore, there is a possibility that grand multiparous women had a higher body mass index and a higher prevalence of gestational diabetes also in Japan, which resulted in the higher prevalence of LGA infant and macrosomia among grand multiparous women. Moreover, access to healthcare and utilization of prenatal care are other possible contributing factors for the association between grand multiparity and adverse birth outcomes. Studies indicated that grand multiparous women tended not to use prenatal care [22, 48], and high parity was associated with late initiation of antenatal care in studies in Sudan and Saudi Arabia [49, 50].

Regarding the sociodemographic factors associated with grand multiparity, advanced parental ages were associated with a higher prevalence of grand multiparity, which was consistent with the findings of a previous study conducted in the United Arab Emirates and Australia [18, 21]. In addition, the household occupation and parental occupations were shown to be associated with it, as mentioned before. Grand multiparity was shown to be associated with lower socioeconomic status in Nigeria and Australia [19, 21], and it was consistent with the results of this study. In addition, non-Japanese maternal status was significantly positively associated with grand multiparity. The fertility rate in Japan was lower than the global average [51]; therefore, it is considered that the proportion of Japanese women with high parity was lower than that of non-Japanese women with high parity. Moreover, the prevalence of grand multiparity increased from 2010 to 2020, and the year was significantly associated with the prevalence. It is known that the prevalence of grand multiparity slightly increased in the periods in Japan [4]. As the result showed, the number of births from primiparous and non-grand multiparous women decreased, and the decreasing number of births from mother with lower parity contributed to an increase in the prevalence of grand multiparity. Although the reason for the increasing number of births from grand multiparous women is not certain, it has been suggested that fertility among grand multiparous women tends not to be influenced by the trend of declining birth rates in Japan.

It was shown in this study that grand multiparity was significantly associated with some of the adverse birth outcomes. It will be meaningful to investigate the underlying mechanisms of these associations in future studies. By taking into account factors such as body mass index and pregnancy complications, we could better understand the reasons for the associations. In addition, it will be meaningful to take into account grand multiparity when assessing the risk of adverse birth outcomes of pregnant women in Japan. In Japan, parity is commonly classified into primiparity and multiparity, probably because of low prevalence of women with higher parity, while it was suggested to be better to classify multiparous women into grand multiparous women and non-grand multiparous women in large-scale studies in the future. In contrast, the absolute risk differences between grand multiparous women and non-grand multiparous women for the adverse birth outcomes were estimated to be approximately 1–3% in this study. Because the prevalence of adverse birth outcomes is relatively low in Japan compared with that in other countries [52–54], the absolute risk differences were not large. Particularly, the prevalence of macrosomia is relatively small in Japan, and the absolute risk difference was below 1%. In contrast, the absolute risk differences increase among mothers with higher prevalence of the adverse birth outcomes, and it is considered that enhanced care for grand multiparous women is needed in high-risk populations, such as older mothers and those with lower socioeconomic status.

One limitation of this study is that information on income and level of education was not available in the data. By taking into account these factors, we could better understand the association between grand multiparity and socioeconomic status. In addition, information on planned or unplanned pregnancies, birth spacing, and use of prenatal care was also unavailable. Short birth spacing and unplanned pregnancies are associated with higher parity [55, 56], and there are studies showing an association between grand multiparous women and lower prenatal care use [22, 48]. By taking into account these factors, we could better understand the reasons for the association of grand multiparity with adverse birth outcomes and sociodemographic characteristics. In contrast, the strength of this study is that we used the official statistics data, and our results reflect the reality of the entire nation of Japan. In addition, the data on all live births were used in the analysis, and the study population was large compared with most of the previous studies in other countries.

## Conclusions

Grand multiparous women were found to have a higher risk of preterm birth, low birth weight, macrosomia, and LGA infant compared with non-grand multiparous women. In addition, households with at least one full-time worker at a larger company and upper non-manual workers had a significantly lower prevalence of grand multiparity that the other household occupation and parental occupations, respectively, suggesting that grand multiparity was associated with socioeconomic status in Japan.

## Supplementary Information

Supplementary Material 1.

## Data Availability

The data that support the findings of this study are available from the Ministry of Health, Labour and Welfare and the National Statistics Center in Japan, while the data were used under license for the current study and are not publicly available. Data are however available from the Ministry of Health, Labour and Welfare and the National Statistics Center if those organizations permit use of the data.

## Abbreviations

CI: Confidence intervals
GVIF: Generalized variance inflation factor
LGA: Large-for-gestational-age
PR: Prevalence ratio
RR: Risk ratio
SGA: Small-for-gestational-age
