# Supplementary material for: Association of grand multiparity with adverse birth outcomes and sociodemographic characteristics: an analysis of nationwide birth data in Japan
Source: Reprod Health. 2025 Dec 27;23:26. doi: 10.1186/s12978-025-02246-0 (PMC12853663; doi:10.1186/s12978-025-02246-0)
Supplement: Supplementary file 1 — Supplementary Material 1. [file 12978_2025_2246_MOESM1_ESM.pdf]

Supplementary table 1. Proportion of missing data for each variable

Variable	Proportion of missing data (%)
Parity	0.00
Maternal nationality	0.00
Paternal nationality	0.00
Maternal age group	0.00
Paternal age group[1]	–
Maternal occupation	3.69
Paternal occupation	4.00
Household occupation	2.21
Infant sex	0.00
Gestational weeks	0.01
Birth weight	0.01
Status of SGA and LGA	0.03

SGA, small-for-gestational-age; LGA, large-for-gestational-age

[1]Because the number of missing data was very small, it was not allowed to report here.

Supplementary table 2. Result of the adjusted regression analysis investigating the associations between parity and adverse birth outcomes using multiple imputation

Parity and adverse birth outcomes[1,2]	Adjusted RR (95% CI)[3]	p-value
Preterm birth		
Primiparous	1.01 (1.00, 1.02)	0.154
Non-grand multiparous	1.00 (Reference)	
Grand multiparous	1.47 (1.40, 1.53)	<0.001
Low birth weight		
Primiparous	1.27 (1.26, 1.28)	<0.001
Non-grand multiparous	1.00 (Reference)	
Grand multiparous	1.25 (1.20, 1.30)	<0.001
Macrosomia		
Primiparous	0.70 (0.69, 0.72)	<0.001
Non-grand multiparous	1.00 (Reference)	
Grand multiparous	1.73 (1.57, 1.90)	<0.001
SGA		
Primiparous	1.06 (1.05, 1.07)	<0.001
Non-grand multiparous	1.00 (Reference)	
Grand multiparous	0.98 (0.94, 1.03)	0.504
LGA		
Primiparous	1.22 (1.21, 1.23)	<0.001
Non-grand multiparous	1.00 (Reference)	
Grand multiparous	1.32 (1.28, 1.37)	<0.001

RR, risk ratio; CI, confidence interval; SGA, small-for-gestational-age; LGA, large-for-gestational-age

[1]Parity was categorized into primiparous (1 delivery), non-grand multiparous (2–4 deliveries), and grand multiparous (≥ 5 deliveries).

[2]Preterm birth was defined as infants born before 37 completed weeks of gestation. Low birth weight was defined as infants weighing <2,500 g, and macrosomia was defined as a birth weight of ≥4,000 g. SGA infants and LGA infants were defined as infants whose birthweights were less than the 10th percentile and greater than the 90th percentile for each combination of gestational age, sex, and parity (primiparity and multiparity), respectively.

[3]Parental age groups, parental nationalities, household occupation, parental occupations, and birth year were adjusted.

Supplementary table 3. Result of the adjusted regression analysis investigating the sociodemographic characteristics of grand multiparity using multiple imputation

Characteristics	Adjusted PR (95% CI)	p-value
Maternal age group		
24 years or less	0.07 (0.05, 0.08)	<0.001
25–29 years	0.42 (0.40, 0.44)	<0.001
30–34 years	1.00 (Reference)	
35–39 years	1.81 (1.75, 1.87)	<0.001
40 years or more	2.89 (2.76, 3.02)	<0.001
Paternal age group		
24 years or less	1.10 (0.95, 1.27)	0.217
25–29 years	0.94 (0.89, 1.00)	0.048
30–34 years	1.00 (Reference)	
35–39 years	1.22 (1.18, 1.27)	<0.001
40 years or more	1.61 (1.55, 1.68)	<0.001
Maternal nationality		
Japanese	1.00 (Reference)	
Non-Japanese	1.29 (1.20, 1.40)	<0.001
Paternal nationality		
Japanese	1.00 (Reference)	
Non-Japanese	0.96 (0.88, 1.04)	0.309
Household occupation[1]		
Farmer	2.70 (2.46, 2.96)	<0.001
Self-employed	3.07 (2.94, 3.20)	<0.001
Full-time worker at a smaller company	1.92 (1.86, 1.99)	<0.001
Full-time worker at a larger company	1.00 (Reference)	
Others	2.52 (2.41, 2.63)	<0.001
Maternal occupation[2]		
Upper non-manual workers	1.00 (Reference)	
Lower non-manual workers	1.57 (1.49, 1.65)	<0.001
Manual workers	2.01 (1.84, 2.19)	<0.001
Other workers	1.79 (1.61, 1.98)	<0.001
Unemployed persons	2.17 (2.06, 2.28)	<0.001
Paternal occupation[2]		

Upper non-manual workers	1.00 (Reference)	
Lower non-manual workers	1.07 (1.03, 1.12)	0.001
Manual workers	2.43 (2.34, 2.53)	<0.001
Other workers	1.42 (1.33, 1.51)	<0.001
Unemployed persons	3.24 (3.01, 3.49)	<0.001
Birth year (fiscal year)		
2010	1.00 (Reference)	
2015	1.02 (0.99, 1.05)	0.300
2020	1.34 (1.29, 1.38)	<0.001

PR, prevalence ratio; CI, confidence interval

[1]Household occupation indicates the type of occupation or employment status of the top earner of the household. The full-time worker at a smaller company indicates a full-time worker at a company with <100 employees, and the full-time worker at a larger company indicates public servants, board member of a company, and full-time workers at a company with ≥100 employees.

[2]Parental occupations were classified into upper non-manual workers (administrative and managerial workers; professional and engineering workers), lower non-manual workers (clerical workers; sales workers; and service workers), manual workers (manufacturing process workers; transport and machine operating workers; construction and mining workers; and carrying, cleaning, packaging, and related workers), other workers (security workers; agriculture, forestry, and fishery workers; and workers in unclassifiable occupations), and unemployed persons in the analysis.
